# Synergistic Effect of Static Compliance and D-dimers to Predict Outcome of Patients with COVID-19-ARDS: A Prospective Multicenter Study

**DOI:** 10.3390/biomedicines9091228

**Published:** 2021-09-15

**Authors:** Tommaso Tonetti, Giacomo Grasselli, Paola Rucci, Francesco Alessandri, Alessio Dell’Olio, Annalisa Boscolo, Laura Pasin, Nicolò Sella, Chiara Mega, Rita Maria Melotti, Massimo Girardis, Stefano Busani, Giacomo Bellani, Giuseppe Foti, Domenico Luca Grieco, Vittorio Scaravilli, Alessandro Protti, Thomas Langer, Luciana Mascia, Francesco Pugliese, Maurizio Cecconi, Roberto Fumagalli, Stefano Nava, Massimo Antonelli, Arthur S. Slutsky, Paolo Navalesi, Antonio Pesenti, Vito Marco Ranieri

**Affiliations:** 1 Dipartimento di Scienze Mediche e Chirurgiche (DIMEC), Anesthesia and Intensive Care Medicine, IRCCS Policlinico di Sant’Orsola, Università di Bologna, 40138 Bologna, Italy; alessio.dellolio@unibo.it (A.D.); chiara-mega@hotmail.com (C.M.); ritamaria.melotti@unibo.it (R.M.M.); m.ranieri@unibo.it (V.M.R.); 2 Fondazione IRCCS Ca’Granda Ospedale Maggiore Policlinico, University of Milan, 20122 Milan, Italy; giacomo.grasselli@unimi.it (G.G.); vittorio.scaravilli@gmail.com (V.S.); antonio.pesenti@unimi.it (A.P.); 3 Department of Pathophysiology and Transplantation, University of Milan, 20122 Milan, Italy; 4 Dipartimento di Scienze Biomediche e Neuromotorie (DIBINEM), Alma Mater Studiorum Università di Bologna, 40126 Bologna, Italy; paola.rucci2@unibo.it (P.R.); luciana.mascia@unibo.it (L.M.); 5 Department of Anaesthesia and Intensive Care, Sapienza University of Rome, 00161 Rome, Italy; francescoalessandri1@yahoo.it (F.A.); f.pugliese@uniroma1.it (F.P.); 6 Anesthesia and Critical Care, Department of Medicine-DIMED, University Hospital of Padua, 35127 Padua, Italy; annalisa.boscolo@gmail.com (A.B.); laurapasin1704@gmail.com (L.P.); nico.sella@hotmail.it (N.S.); paolo.navalesi@unipd.it (P.N.); 7 Anesthesia and Critical Care, Policlinico di Modena, Università di Modena e Reggio Emilia, 41125 Modena, Italy; girardis@unimore.it (M.G.); stefano.busani@unimore.it (S.B.); 8 Department of Medicine and Surgery, University of Milano-Bicocca, 20900 Monza, Italy; giacomo.bellani1@unimib.it (G.B.); g.foti@asst-monza.it (G.F.); thomas.langer@unimib.it (T.L.); roberto.fumagalli@ospedaleniguarda.it (R.F.); 9 Department of Anesthesia and Intensive Care Medicine, ASST Monza—Ospedale San Gerardo, 20900 Monza, Italy; 10 Department of Anesthesiology and Intensive Care Medicine, Fondazione Policlinico Universitario A. Gemelli IRCCS, 00168 Rome, Italy; dlgrieco@outlook.it (D.L.G.); massimo.antonelli@policlinicogemelli.it (M.A.); 11 Department of Biomedical Sciences, Humanitas University, 20089 Pieve Emanuele, Italy; alessandro.protti@hunimed.eu (A.P.); maurizio.cecconi@hunimed.eu (M.C.); 12 Humanitas Clinical and Research Center—IRCCS, 20089 Rozzano, Italy; 13 Dipartimento di Anestesia e Rianimazione Grande Ospedale Metropolitano Niguarda, 20162 Milan, Italy; 14 Respiratory and Critical Care Unit, Department of Clinical, Integrated and Experimental Medicine (DIMES), S. Orsola-Malpighi Hospital, Alma Mater University, 40138 Bologna, Italy; stefano.nava@unibo.it; 15 Keenan Research Centre for Biomedical Science, St Michael’s Hospital, Toronto, ON M5B 1W8, Canada; arthur.slutsky@unityhealth.to

**Keywords:** acute respiratory distress syndrome, COVID-19, D-dimer, static compliance, mechanical ventilation

## Abstract

The synergic combination of D-dimer (as proxy of thrombotic/vascular injury) and static compliance (as proxy of parenchymal injury) in predicting mortality in COVID-19-ARDS has not been systematically evaluated. The objective is to determine whether the combination of elevated D-dimer and low static compliance can predict mortality in patients with COVID-19-ARDS. A “training sample” (March–June 2020) and a “testing sample” (September 2020–January 2021) of adult patients invasively ventilated for COVID-19-ARDS were collected in nine hospitals. D-dimer and compliance in the first 24 h were recorded. Study outcome was all-cause mortality at 28-days. Cut-offs for D-dimer and compliance were identified by receiver operating characteristic curve analysis. Mutually exclusive groups were selected using classification tree analysis with chi-square automatic interaction detection. Time to death in the resulting groups was estimated with Cox regression adjusted for SOFA, sex, age, PaO_2_/FiO_2_ ratio, and sample (training/testing). “Training” and “testing” samples amounted to 347 and 296 patients, respectively. Three groups were identified: D-dimer ≤ 1880 ng/mL (LD); D-dimer > 1880 ng/mL and compliance > 41 mL/cmH_2_O (LD-HC); D-dimer > 1880 ng/mL and compliance ≤ 41 mL/cmH_2_O (HD-LC). 28-days mortality progressively increased in the three groups (from 24% to 35% and 57% (training) and from 27% to 39% and 60% (testing), respectively; *p* < 0.01). Adjusted mortality was significantly higher in HD-LC group compared with LD (HR = 0.479, *p* < 0.001) and HD-HC (HR = 0.542, *p* < 0.01); no difference was found between LD and HD-HC. In conclusion, combination of high D-dimer and low static compliance identifies a clinical phenotype with high mortality in COVID-19-ARDS.

## 1. Introduction

Patients hospitalized for coronavirus disease-2019 (COVID-19) may develop severe hypoxemia requiring Intensive Care Unit (ICU) admission and mechanical ventilation. Acute Respiratory Distress Syndrome (ARDS), the most severe form of hypoxic respiratory failure, occurs in about 15% to 68% of hospitalized COVID-19 patients [1], and is characterized by vascular thrombosis [2] and loss of lung aeration [3]. Studies performed in patients with ARDS from other cause than COVID-19 (“classical ARDS”) have demonstrated that D-dimers are a proxy of intra-alveolar coagulation and fibrinolysis [4] and static compliance of the respiratory system is a proxy of the size of the ventilable lung (“baby lung”) [5].

Extension of vascular thrombosis [6,7] and amount of loss of lung aeration [8,9] has been correlated to clinical outcome of severe COVID-19. Consistently with these data, several studies performed in patients with COVID-19 ARDS showed that (a) concentrations of D-dimers were higher in non-survivors than in survivors [10,11,12,13,14,15]; (b) lower compliance of the respiratory system in the first day of ventilation was associated with increased risk of 28-day mortality [16]. However, the bulk of these data are not conclusive with respect to the importance of D-dimers and compliance in predicting outcomes in patients with COVID-19 ARDS since (a) most studies were retrospective in nature [10,11,12,13,14]; (b) a synergistic effect of D-dimers and compliance was observed but was not thoroughly analysed; (c) a prognostic model based on D-dimers and compliance was not validated using rigorous statistical techniques based on different samples [17].

The objective of the present study is to prove in two separate samples (a “training sample” and a “testing sample”) of patients with COVID-19 ARDS the hypothesis that only the combination of elevated plasmatic D-dimers and reduced respiratory system compliance may predict mortality in patients with COVID-19-ARDS.

## 2. Methods

Data were prospectively collected from nine Italian hospitals (Policlinico di Sant’Orsola (Alma Mater Studiorum, Università di Bologna), Policlinico di Modena (Università di Modena e Reggio Emilia), Ospedale Maggiore Policlinico (Università di Milano), Ospedale Niguarda (Università di Milano-Bicocca, Milan), Ospedale San Gerardo di Monza, (Università di Milano-Bicocca), Istituto Clinico Humanitas (Università Humanitas, Milano), Azienda Ospedaliero-Universitaria (Università di Padova), Policlinico Gemelli (Università Cattolica del Sacro Cuore, Roma), Policlinico Umberto I (Sapienza Università di Roma) Roma). Institutional Review Boards at each hospital approved the study protocol and consent was obtained according to local indications [18]. Patients older than 18 years with confirmed COVID-19 [11] who were admitted to the ICUs were enrolled. Diagnosis of ARDS according to the Berlin definition [19] and invasive mechanical ventilation within 24 h after admission were the inclusion criteria. A “training sample” during the period March–June 2020 and a “testing sample” during the period September 2020–January 2021 of the pandemic were collected [20]. Study outcome was all-cause mortality at 28-days. The first available values of D-dimer and static compliance of the respiratory system during the first 24 h from study admission were recorded. Static compliance was calculated as previously described [21,22]. End-inspiratory plateau pressure and total positive end-inspiratory pressure were obtained by performing end-inspiratory and end-expiratory occlusions with patients sedated, paralyzed and ventilated in volume-control mode [21,22].

### Statistical Methods

Receiver operating characteristic (ROC) curve analysis was used to identify the optimal cut-off that balanced sensitivity and specificity for D-dimer and static compliance in predicting 28-day mortality [17]. The identified cut-off values of D-dimer and static compliance were then used to perform a classification tree analysis (CTA) with chi-square automatic interaction detection (CHAID) [23,24,25]. The CTA procedures build decision trees beginning with a root node that includes all cases, then the tree branches into subgroups (or nodes) and grows iteratively. The best discriminating predictor is selected first, and then subsequent predictors are entered into the procedure if they contribute significantly to subtyping cases into homogeneous groups. Variables not useful in discriminating cases do not enter the procedure. The tree grows until a stopping criterion is met or no further significant improvement in the classification of study participants is possible. At the end of the procedure, the study population is partitioned into terminal nodes that are as homogeneous as possible with respect to the categories of the dependent variable [23,24,25]. The dichotomized D-dimer and static compliance variables were used as input for the CTA procedure. As a rule, the classification tree should be derived in one sample and validated in a separate sample. We chose to use the two different time periods for the derivation (training) and validation (testing) sample.

Cox regression analysis was used to predict time to death at 28-days as a function of the groups resulting from the CTA procedure, using the group with highest mortality as class reference, and adjusting for sequential organ failure assessment (SOFA) score at admission, sex, age, PaO_2_/FiO_2_ ratio [18,26], and sample (training/testing).

Continuous variables were expressed as medians and IQRs. Categorical variables were summarized as absolute and percentage frequencies. Comparison of continuous data between samples was done using Mann-Whitney or Kruskal-Wallis test and comparison of categorical data was done using χ^2^ or Fisher’s exact test. All statistical tests were two sided. The significance level was set at *p* < 0.05 and no imputation of missing data was performed. Analyses were done using IBM SPSS (IBM Corp. Released 2019. IBM SPSS Statistics for Windows, Version 26.0. IBM Corp., Armonk, NY, USA).

## 3. Results

Seven-hundred and thirty patients were screened. Eighty-seven patients were excluded (36 because they did not match ARDS criteria, and 51 for missing values of D-dimers and static compliance on admission). Of the remaining 643 patients, 347 were admitted from March–June 2020 (“training sample”) and 296 from September 2020–January 2021 (“testing sample”).

In the overall study cohort (643 patients), median time from hospital admission to intubation was 3 days (IQR 1–5). Median age was 64 years (56–71), 530 (77.4%) were males, and all were ventilated according to a conventional protective ventilatory strategy [21]. Median static compliance was 41 mL/cmH_2_O (33–52) and median D-dimer concentration was 1560 ng/mL (704–4900). Table 1 shows baseline characteristics of the “training” and “testing” samples. Small but significant difference in SOFA score at admission, PaO_2_/FiO_2_, static compliance, and ventilator settings were observed. The 28-day mortality was 36.8% (143 of 389 patients) in the training sample, and 37.2% (110 of 296) in the testing sample (χ^2^ = 0.012, *p* = 0.914).

**Figure 1:**
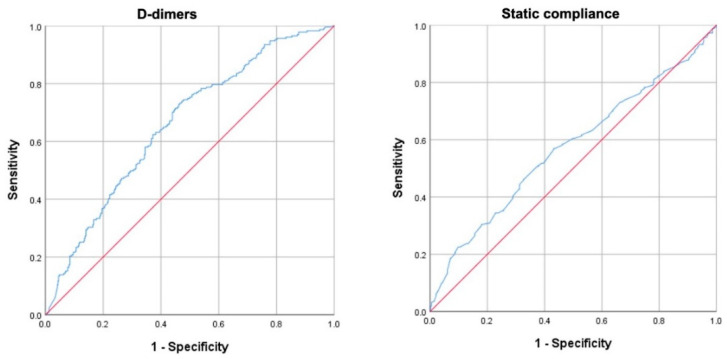
ROC curves for D-dimers (**left** panel) and compliance of the respiratory system (**right** panel). The actual ROC curves are blue, while the red line represents an arbitrary (theoretical) test that is expected a priori to have no discriminatory value.

The areas under the ROC curves in all 643 patients for D-dimers and static compliance were both significantly larger than that of an arbitrary test without a discriminatory value (AUC = 0.657; 95% CI 0.614–0.700 and AUC = 0.580; 95% CI 0.533–0.628, respectively) (Figure 1**)**. The cut-off values for D-dimers and static compliance that balanced sensitivity and specificity were 1880 ng/mL (sensitivity 61.9%; specificity 63.6%) and 41 mL/cmH_2_O (sensitivity 58.0%; specificity 56.6%).

**Figure 2:**
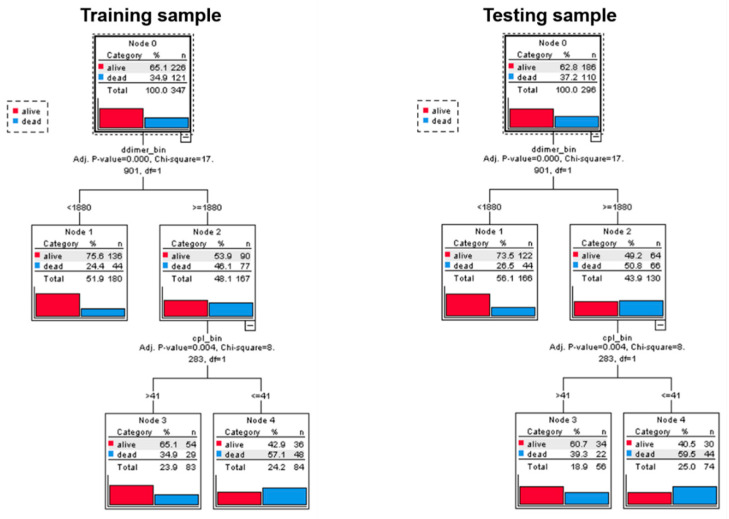
Classification tree results in the training (N = 347) and in the testing (N = 296) sample. Both trees have three final nodes.

Classification tree analysis partitioned the study population into three mutually exclusive groups: patients with D-dimer ≤ 1880 ng/mL (LD); patients with D-dimer > 1880 ng/mL and static compliance > 41 mL/cmH_2_O (HD-HC); patients with D-dimer > 1880 ng/mL and compliance ≤ 41 mL/cmH_2_O (HD-LC). The probability of death at 28-days progressively increased, from 24% to 35% and to 57% in the training sample and from 27% to 39% and 60% in the testing sample, respectively (χ^2^ = 17.901, *p* < 0.001 at the first partition and χ^2^ = 8.283, *p* = 0.004 at the second partition) (Figure 2).

**Figure 3:**
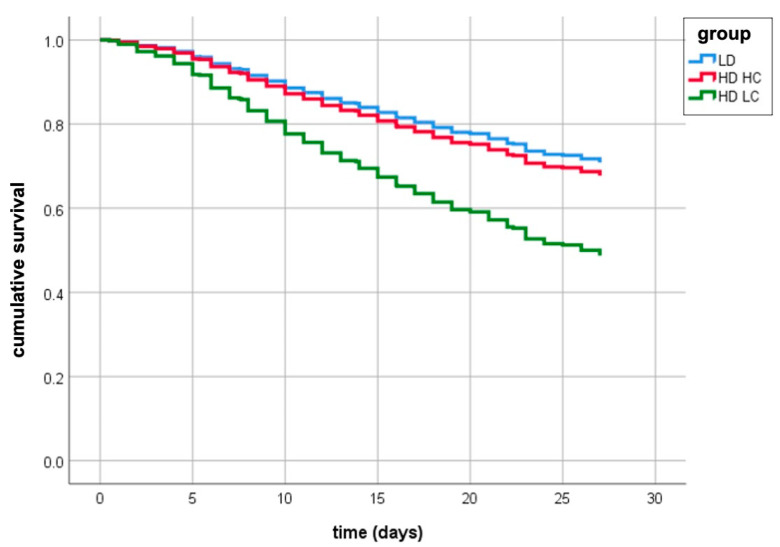
Survival estimates of patients with low D-dimer (LD), high-D-dimer and low compliance (HD-LC), high-D-dimer and high compliance (HD-HC) from Cox regression analysis, adjusted for age, gender, SOFA, PaO_2_/FiO_2_ ratio, and sample (testing vs. training).

Cox regression analysis demonstrated that mortality, adjusted for covariates (age: HR = 1.075, 95% CI 1.058–1.092, *p* < 0.001; SOFA: HR = 1.084, 95% CI 1.015–1.158; PaO_2_/FiO_2_ ratio: HR = 0.995, 95% CI 0.993–0.998), was significantly higher in the HD-LC group compared with the LD (HR = 0.479, 95% CI 0.356–0.647, *p* < 0.001) and the HD-HC (HR = 0.542, 95% CI 0.380–0.772, *p* < 0.01); no difference in mortality was found between LD and HD-HC (Table 2 and Figure 3).

## 4. Discussion

The present study shows that in patients with COVID-19-ARDS combination of baseline plasma concentrations of D-dimer higher than 1880 ng/mL and of baseline values of respiratory system compliance lower than 41 mL/cmH_2_O are associated with a significantly increased risk of death at 28-days, compared to patients presenting with alterations in only one of each parameter.

Mortality rate of hospitalized COVID-19 patients ranges between 13% and 89% [1] with an associated relative risk of death of 7.99 (95% CI: 4.9 to 13) [27]. Pathophysiology of COVID-19 ARDS is characterized by loss of lung aeration with large consolidated, non-aerated regions and ground-glass opacities [28] and by platelet-fibrin microthrombi at the alveolar-capillary barrier [29,30,31]. Since proportion of compromised lung volume [3,8] and abnormal coagulation parameters [7] have been associated with outcome, several studies have investigated the role of static compliance [9,16,18], and D-dimers [32] to predict outcome. This is based on several studies performed in “classical ARDS” that have demonstrated that static compliance is a reasonable proxy of the size of the normally aerated lung (baby lung) [5] and that D-dimers are a marker of thromboembolic disorders and extravascular fibrin deposition [4].

Early descriptions of COVID-19 demonstrated that increased concentrations of D-dimers were associated with worse clinical outcome [10,11,12,13,14,15]. A cohort study including 2377 adults hospitalized with COVID-19 in a large New York City hospital network, showed that patients presenting with elevated baseline concentration of D-dimer within “normal” ranges were less likely to have critical illness (odds ratio 2.4) [32]. A multi-center cohort study collected data by chart review of 3418 ICU patients and showed that more than 93% of patients had D-dimers values above the upper normality limit and an almost two-fold increased odds of death was observed in the patients with D-dimer levels higher than ~4000 ng/mL [33]. A systematic review of more than 2700 patients with COVID-19 showed that patients with increased D-dimers had an odds ratio greater than 5 for developing severe disease [34]. Three retrospective studies on COVID-19 hospitalized patients provide ROC analysis on 248, 343 and 1065 consecutive COVID-19 cases, respectively. Plasma D-dimer concentrations >2140 ng/mL, >2000 ng/mL and >2380 ng/mL at admission were respectively identified as the optimal cut-offs for discriminating survivors from non-survivors (AUC = 0.85, 0.89 and 0.69, respectively) with sensitivities of 88.2%, 92.3% and 51%, respectively; specificities were 71.3%, 83.3% and 78%, respectively [35,36,37].

Data regarding the association between static compliance and outcome in patients with COVID-19 ARDS are less consistent. Botta et al. in a retrospective study of 553 patients found that lower static compliance on the first day of ventilation was associated with increased risk of death at 28-day (OR 0.75 (95% CI 0.57–0.98), *p* = 0.037) [16]. The French REVA network found a small but significant difference in static compliance between survivors and non-survivors ((34 (27–43) vs. 32 (24–41) mL/cmH_2_O; *p* < 0.001)) [38]. On the contrary, Vandenbunder et al. in a prospective study including 372 patients found that static compliance on the first day of ventilation was not related with 28-day survival [9]. Moreover, a sensitivity analysis of 742 patients with COVID-ARDS showed that ICU discharge and risk of death at 28-days were not influenced by static compliance [39]. Grasselli et al. examined the relationship of baseline D-dimer and static compliance with mortality. In 301 patients with COVID-19-ARDS, patients with static compliance less than/equal to the observed median (41 mL/cmH_2_O) and D-dimer concentrations greater than the median (1880 ng/mL) had markedly increased 28-day mortality compared with other patients [18]. Interestingly, the cut-off values identified by ROC curves in the present study very similar to the median values of the study by Grasselli et al. [18]; this may be due to the fact that all patients from that study are included in the “training set” of the present study. Although the present study may appear in some ways similar to the previous study from our group, we believe the present study conveys a more methodologically robust message and allows for more definitive conclusions on the role of D-dimers and static compliance in the pathophysiology and outcome of COVID-19-ARDS patients.

However, the bulk of these studies remain inconclusive regarding the impact of D-dimers and static compliance in predicting outcome in patients with COVID-19 ARDS because of the retrospective nature of most studies and because they did not use rigorous statistical techniques to evaluate the predictive ability of these variables in independent samples [10,11,12,13,14,18].

The major strength of the present study is the use of robust statistical methods to identify threshold values of D-dimer (1880 ng/mL) and static compliance (41 mL/cmH_2_O) associated with the risk of death with optimal sensitivity and specificity through ROC analysis. We used these cut-off values to stratify patients into subgroups with different mortality risk through classification tree analysis and validated this tree in an independent sample. Specifically, classification tree analysis generated three mutually exclusive groups: patients with D-dimer ≤ 1880 ng/mL (LD); patients with D-dimer > 1880 ng/mL and static compliance > 41 mL/cmH_2_O (HD-HC); patients with D-dimer > 1880 ng/mL and static compliance ≤ 41 mL/cmH_2_O (HD-LC). Probability of death at 28-days progressively increased in these three groups in both the training and testing datasets. Interestingly, static compliance did not contribute significantly to subtyping patients with low D-dimers and was therefore excluded from the respective branch. Lastly, Cox regression analysis showed that, after adjusting for covariates (age, SOFA, and PaO_2_/FiO_2_ ratio), mortality was significantly higher in the HD-LC group compared with the LD and the HD-HC; no difference in outcome was observed between LD and HD-HC (Table 2 and Figure 3).

This study has several limitations. First, it should be acknowledged that other laboratory parameters (e.g., LDH, lymphocytes, creatinine, C-reactive protein) have been associated with severity and mortality of COVID-19 [40,41,42]. However, since we aimed to identify predictors of outcome in COVID-19-ARDS (and not in COVID-19), we chose to focus on D-dimers and static compliance based on the preliminary observation that only the combination of increased D-dimers and low static compliance (rather than high D-dimers only vs. low compliance only) identifies a specific phenotype of COVID-19-ARDS patients characterized by very high mortality [18]. Second, although D-dimers are considered a sensitive biomarker for thromboembolic disorders and extravascular fibrin deposition [4,43], and previous studies suggested that high concentration of D-dimer in the broncho-alveolar lavage of patients with classic ARDS reflect thrombotic activity and fibrin degradation [44], several studies have shown that D-dimers are not specific since other conditions such as pregnancy, renal failure, sepsis are associated with raised D-dimer levels, and that plasma D-dimer levels can be caused by lysis of extra-vascular rather than intra-vascular fibrin [45,46,47]. Moreover, the relatively “small” areas under the ROC curves (0.657; 95% CI 0.614–0.700 for D-dimers, and 0.580; 95% CI 0.533–0.628, for static compliance) is another potential limitation of the study; further, AUC for D-dimers is lower than in two previous studies [35,36], but very similar to another, bigger, retrospective study [37] and this could be due, at least in part, to the intrinsic heterogeneity of consecutive ICU patients. However, classification tree analysis may overcome these limitations since ROC analysis was implemented to objectively identify cut-off values that were validated by the classification tree analysis with chi-square automatic interaction detection [23,24,25]. Finally, although low values of static compliance largely reflect the degree of lung volume loss [5], when evaluated by the Berlin definition as criterion for severity of ARDS, compliance was not able to identify a group of patients with higher mortality [19].

In conclusion, the present study demonstrates that, in mechanically ventilated patients with COVID-19-ARDS, only the combination D-dimer values higher than 1880 ng/mL and compliance of the respiratory system less than 41 mL/cmH_2_O allow patient stratification into subgroups at increased risk of death, and identify a clinical phenotype with extremely high mortality that may benefit from more aggressive treatment and may be included in future trials based on enrichment strategies.

## Figures and Tables

**Table 1:** Demographic, ventilatory and laboratory variables collected within 24 h of ICU admission in COVID-19-ARDS patients.

	Training Sample	Testing Sample	*p*-Value
Male gender (*n* (%))	302 (77.6)	228 (77.0)	0.8506
Age (years)	64 (56–70)	65 (57–71)	0.3228
Time from hospital admission to invasive mechanical ventilation (days)	2 (1–5)	3 (1–7)	0.1117
SOFA score at ICU admission	4 (4–6)	4 (3–5)	<0.0001

Weight (kg)	85 (75–92)	85 (75–95)	0.6206
Height (cm)	171 (168–178)	170 (165–178)	0.5421
BMI (kg/m^2^)	27.8 (25.6–31.1)	27.8 (26.0–31.3)	0.2610
PBW (kg)	66 (62–73)	66 (61–73)	0.5473

Respiratory rate (bpm)	20 (16–24)	19 (16–22)	0.1704
P/F ratio (mmHg)	132 (94–176)	114 (86–150)	0.0003
PEEP (cmH_2_O)	12 (10–14)	10 (10–12)	<0.0001
Tidal volume (mL)	480 (420–530)	450 (400–500)	0.0001
TV/PBW (mL/kg)	7.1 (6.4–8.1)	6.8 (6.3–7.6)	0.0077
Plateau pressure (cmH_2_O)	24 (22–27)	23 (21–25)	<0.0001
Static compliance of the respiratory system (mL/cmH_2_O)	42 (34–53)	40 (31–49)	0.0041
pH (units)	7.39 (7.33–7.43)	7.38 (7.33–7.44)	0.7407
PaO_2_ (mmHg)	82 (70–104)	85 (72–107)	0.0581
PaCO_2_ (mmHg)	46 (39–53)	44 (38–51)	0.2559
D-dimer (ng/mL)	1620 (714–5111)	1510 (669–4685)	0.5209

Glucocorticoids (*n* (%))	145/336 (43.2)	243/296 (82.1)	<0.0001
Full-dose anticoagulation (*n* (%))	213/317 (67.2)	244/291 (83.8)	<0.0001
Remdesivir (*n* (%))	66/270 (24.4)	34/296 (11.5)	0.0001
Tocilizumab (*n* (%))	67/274 (24.5)	0/296 (0.0)	<0.0001
Hydroxychloroquine (*n* (%))	293/305 (96.1)	0/296 (0.0)	<0.0001

Continuous variables are presented as median (1st–3rd quartile); categorical variables are expressed as absolute number (percentage). Abbreviations: BMI, body mass index; PBW, predicted body weight; P/F ratio, PaO_2_/FiO_2_ ratio; PEEP, positive end-expiratory pressure; TV, tidal volume; PaO_2_, arterial partial pressure of oxygen; PaCO_2_, arterial partial pressure of carbon dioxide.

**Table 2:** Results of Cox proportional risk analysis for mortality. Class reference is HD-LC.

Factor		Hazard Ratio (95% CI)
Class	LD	0.479 (0.356–0.647)
	HD-HC	0.542 (0.380–0.772)
	HD-LC	1.000 (reference)
Age		1.075 (1.058–1.092)
SOFA score		1.084 (1.015–1.158)
P/F ratio		0.995 (0.993–0.998)

HD-LC: High D-dimers-Low Compliance; HD-HC: High D-dimers-High Compliance; LD: Low D-dimers; P/F: arterial to inspiratory oxygen; SOFA score: Sequential Organ Failure Assessment (SOFA) Score.

## Data Availability

De-identified individual participant data that underlie results reported in this article will be available. Applicant must provide: (1) a methodologically sound approach to achieve scientific aims; (2) formal documents of Ethics Committee approval of applicant’s institution. Data will be made available pending authorization of the Policlinico di Sant’Orsola Ethics Committee that will review applicant’s request and after signing an appropriate data sharing agreement. Proposals should be directed to m.ranieri@unibo.it. Data will be available following publication; no end date.
